# On the Passivity of Hysteretic Systems with Double Hysteretic Loops

**DOI:** 10.3390/ma8125465

**Published:** 2015-12-03

**Authors:** Francesc Pozo, Mauricio Zapateiro

**Affiliations:** 1 CoDAlab, Departament de Matemàtiques, Escola Universitària d’Enginyeria Tècnica Industrial de Barcelona (EUETIB), Universitat Politècnica de Catalunya (UPC), Comte d’Urgell, 187, 08036 Barcelona, Spain; 2 EDMA Innova, C. dels Olms 10, 17111 Vulpellac (Girona), Spain; mauricio.zapateiro@edma.cat

**Keywords:** hysteresis, passivity, double-loop, Bouc–Wen model

## Abstract

The Bouc–Wen hysteresis model is widely employed to mathematically represent the dynamical behavior of several physical devices, materials and systems such as magnetorheological dampers, lanthanide zirconium or aluminum oxides, mechanical structures or biomedical systems. However, these mathematical models must account for different properties such as the bounded-input bounded-output stability, asymptotic motion, thermodynamic admissibility or passivity in order to be physically consistent with the systems they represent. The passivity of a system is related to energy dissipation. More precisely, a system is passive if it does not generate energy but only dissipates it. The objective of this paper is to prove that two different double-loop Bouc–Wen models are passive under a particular set of model parameters.

## 1. Introduction

Hysteresis is a characteristic of systems (frequently physical or mechanical systems) that do not immediately follow the forces that are applied to them, but react moderately, or do not recover completely the original state. In other words, hysteretic systems are systems whose states depend on their existing history. The hysteresis phenomenon is present in physical devices, materials and systems such as magnetorheological dampers, lanthanide zirconium or aliminum oxides, electromagnetic servo motors, mechanical structures and biomedical systems [1,2,3,4,5,6,7]. There are several mathematical models that have been proposed in the literature that try to match the observed hysteretic dynamic of the device or system as can be seen, for instance in [8,9], to name a few. The Bouc–Wen model [10] is frequently used to model hysteresis of devices that exhibit this kind of dynamic. However the usual black-box approach employed for determining the parameters of the model according to the experimental data does not necessarily lead to a physically consistent model [11]. Physical consistency is related to properties such as bounded-input bounded-output (BIBO) stability, asymptotic motion, passivity, thermodynamic admissibility, among others. In [12] a comprehensive analysis of BIBO stability, passivity and assymptotic motion was developed for the Bouc–Wen hysteresis model. Furthermore, the thermodynamic admissibility analysis was developed in [13]. Those properties have been experimentally observed and reported accordingly [11].

A special case of hysteresis where double loops were observed have been reported in smart materials such as shape memory alloys ([14,15]), mechanical structural systems such as reinforced concrete structures [16] and lightweight steel shear wall structures [17]. Modeling of asymmetric hysteretic loops behaving as double-loops has been stated in [18], where hysteresis modeling is supported on a modification to a formerly reported model which uses hysteretic cycle decoupling by making the shape parameters system dynamic-dependent, but no position or acceleration information are considered. Moreover, in [19], a model of a kind of double-loop hysteretic behavior is also shown. Again, no position or acceleration is invoked. It is convenient to note that in both models [18,19], the internal dynamic model of hysteresis is converted from first to second order. Traditionally, the internal dynamic of hysteresis model is a first-order differential equation with velocity information supplied as its input, which is the case of the Bouc–Wen model [10].

A mathematical representation based on the Bouc–Wen model of this particular hysteresis behavior was proposed in [20]. In that work, the authors proposed two models incorporating position and acceleration information to the original Bouc–Wen model. The authors analyzed the BIBO stability of both models. In this paper we go a step further and demonstrate the passivity of the double-loop Bouc–Wen model for a particular set of parameters chosen based on the previous works developed by [11,12,13].

This paper is organized as follows. In Section 2 the double-loop Bouc–Wen models using acceleration and position information are revisited. In Section 3 a brief explanation for passivity is presented. Then in Section 3.2 and Section 3.3 the two main passivity theorems are presented, including the development of the passivity proofs. Finally, some conclusions are outlined in Section 4.

## 2. The Double-Loop Bouc-Model Model

Consider a physical system with a hysteretic component that can be represented by a map x(t)→Φ(x)(t). The double-loop Bouc–Wen model using acceleration information was first proposed by Pozo *et al.* [20] as a modification of the original Bouc–Wen model where the acceleration is introduced in the hysteresis differential equation by means of the signum term and is given by:

(1) $$\Phi_{\text{acc}}\left(x\right)\left(t\right)=\alpha kx(t)+(1-\alpha )Dkz(t)$$

(2) $$\begin{array}{cc} \dot{z} & =D^{-1}(A\dot{x}-\beta |\dot{x}|{\left|z\right|}^{n-1}z-\lambda \dot{x}{\left|z\right|}^{n}\\ & +\gamma \mathrm{sgn}\left(\ddot{x}\right)|\dot{x}\left|\right) \end{array}$$

Similarly, the double-loop Bouc–Wen model using position information was also proposed by Pozo *et al.* [20] and is given by:

(3) $$\Phi_{\text{pos}}\left(x\right)\left(t\right)=\alpha kx(t)+(1-\alpha )Dkz(t)$$

(4) $$\begin{array}{cc} \dot{z} & =D^{-1}(A\dot{x}-\beta |\dot{x}{\left||z\right|}^{n-1}z-\lambda \dot{x}{\left|z\right|}^{n}\\ & +\gamma \mathrm{sgn}\left(x\right)|\dot{x}\left|\right) \end{array}$$

In those models, *A*, *β*, *λ* and *γ* are dimensionless parameters that control the shape and the size of the hysteresis loop, D>0, k>0, 0<α<1 and n≥1 is a scalar that governs the smoothness of the transition from elastic to plastic response.

To show the behavior of the double-loop Bouc–Wen model using position information, consider the system

$$\begin{array}{c} m\ddot{x}+c\dot{x}+\Phi \left(x,t\right)=u\left(t\right) \end{array}$$

The restoring force is described as follows:

$$\begin{array}{cc} \Phi (x,t) & =x(t)+z(t),\\ \dot{z} & =\dot{x}-8|\dot{x}\left|z+\text{sgn}\left(x\right)\right|\dot{x}| \end{array}$$

**Figure 1:**
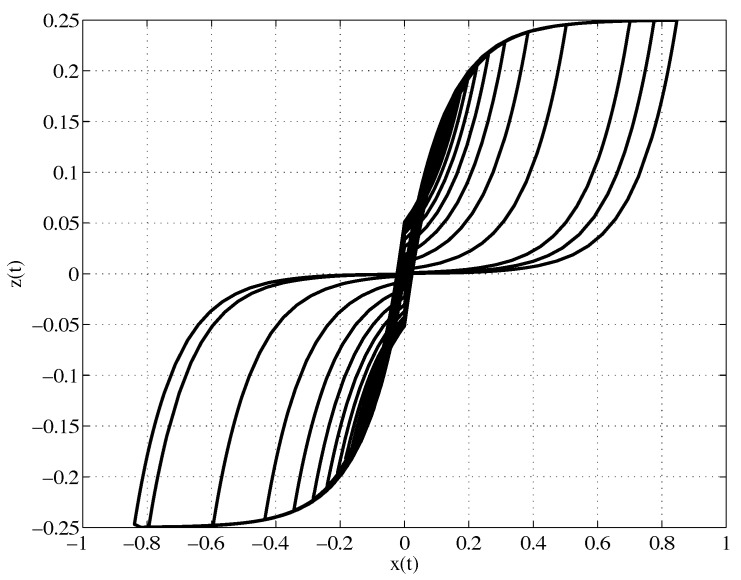
Simulation results. Plot of z(t) *versus* x(t).

The simulation results are presented in Figure 1 where we have set m=c=1 and the forced vibration is given by $u\left(t\right)=\sin\left((0.03t+0.2)\right)$. In this Figure, it can be clearly observed the double-loop behavior of the hysteresis loop. One of the first models that describes this phenomenon was proposed by Baber and Noori [21] in the characterization of pinching behavior under cyclic displacement or to describe hysteretical systems with slip [19].

### 2.1. Bounded-Input Bounded-Output (BIBO) stability

One of the elements of the physical consistency lies in the experimentally based premise that a true physical hysteretic element is BIBO-stable, which means that, for any bounded input signal x(t), the hysteretic response is also bounded. Therefore, the models in Equations (1)–(4) should keep the BIBO stability property in order to considered an adequate candidate to model real physical systems. This motivates the following definition:

**Definition 1.**  *Given the parameters* 0<α<1,k>0,D>0,A,β,λ,γ *with* β+λ≠0 *and* n>1, *the set* Ω *is defined as the set of initial conditions* z(0) *for which the modified Bouc–Wen model in Equations* (1)–(4) *is BIBO stable, respectively.*

The following Theorem can be used as a classification of the BIBO-stable double-loop Bouc–Wen models using acceleration and position information.

**Theorem 1 ([20]).**  *Let* x(t),t∈[0,∞) *be a* $C^{1}$ *input signal and*

$$\begin{array}{cc} z_{0}^{+} & ≜\sqrt[{n}]{\frac{A+\gamma }{\beta +\lambda }},\;z_{0}^{-}≜\sqrt[{n}]{\frac{A-\gamma }{\beta +\lambda }},\;z_{0}≜\sqrt[{n}]{\frac{A+|\gamma |}{\beta +\lambda }},\;z_{0}^{\prime }≜\sqrt[{n}]{\frac{A-|\gamma |}{\beta +\lambda }}\\ z_{1}^{+} & ≜\sqrt[{n}]{\frac{A+\gamma }{\lambda -\beta }},\;z_{1}^{-}≜\sqrt[{n}]{\frac{A-\gamma }{\lambda -\beta }},\;z_{1}≜\sqrt[{n}]{\frac{A-|\gamma |}{\lambda -\beta }},\;z_{1}^{\prime }≜\sqrt[{n}]{\frac{A+|\gamma |}{\lambda -\beta }} \end{array}$$

*Then, the BIBO-stable double-loop Bouc–Wen models in Equations* (1)–(4) *are identified and classified in Table 1.*

From Theorem 1 and Table 1 it can be concluded that when the set Ω is empty (class VI), this means that the double-loop Bouc–Wen model in Equations (1)–(4) is not BIBO stable. Moreover, for each class from I to V –depending on the values of *A*, *β*, *γ* and *λ*– explicit bounds for the hysteretic variable z(t) can be determined.

**Table 1:** Classification of the bounded-input bounded-output (BIBO)-stable double–loop Bouc–Wen models in Equations (1)–(4).

Class	Parameters	Ω	Upper Bound
I	A±γ>0, β+λ>0, β-λ≥0	R	$\max\{|z\left(0\right)|,z_{0}\}$
II	A±γ>0, β-λ<0, β≥0, Aβ-λ|γ|≥0	$\left[-z_{1},z_{1}\right]$	$\max\{|z\left(0\right)|,z_{0}\}$
III	A±γ<0, β-λ>0, β+λ≥0	R	$\max\{|z\left(0\right)|,z_{1}\}$
IV	A±γ<0, β+λ<0, β≥0, Aβ+λ|γ|≤0	$\left[-z_{0}^{\prime },z_{0}^{\prime }\right]$	$\max\{|z\left(0\right)|,z_{1}^{\prime }\}$
V	A±γ, β+λ>0, β-λ≥0	R	|z(0)|
VI	All other cases	∅	

The different classes of the BIBO-stable double-loop Bouc–Wen models in Table 1 are obtained during the proof of Theorem 1 in the work by Pozo *et al.* [20]. In this work, the proof is divided in several cases and subcases leading to different Ω sets of initial conditions for which the system is stable and to different upper bounds. Finally, six different classes are defined.

In the works by [11,12,13] it was proved that only Class I Bouc–Wen single-loop model was physically consistent. With the strategies and results presented in the papers [11,12,13] and due to the analogy between the single and double loop models, we will concentrate on the same case in this paper, proving—in Section 3—the passivity of Class I of both models in Equations (1)–(4).

As a matter of example and to illustrate this fact, consider the free motion of a second order system given by:

(5) $$\ddot{x}+\dot{x}+\Phi_{\text{acc}}\left(x\right)\left(t\right)=0$$

**Figure 2:**
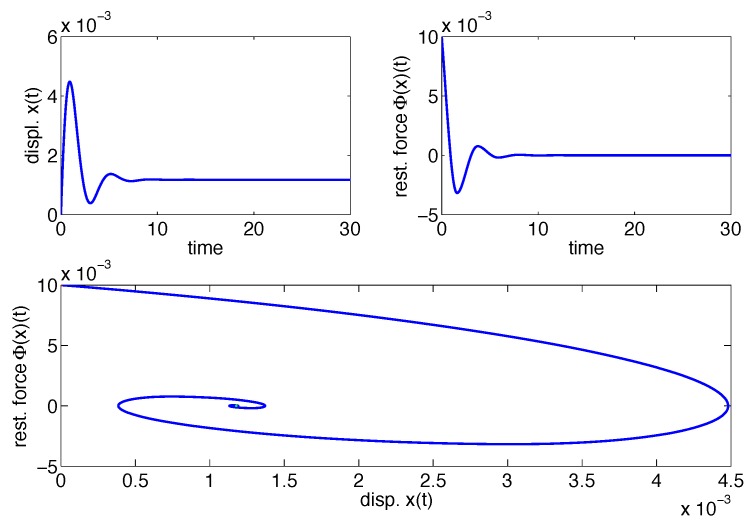
Limit cycle absence in Class I models.

Let us assume that Φ(x)(t) is a Class I model so that A=2, β=4, λ=0, γ=1 and n=1. Initial conditions are set to x(0)=0, $\dot{x}\left(0\right)=0.1$ and z(0)=0. The upper left plot in Figure 2 shows the displacement x(t) of the system described in Equation (5) *versus* the time, the upper right plot shows the restoring force Φ(x)(t) *versus* the time and, finally, the lower plot shows the two-dimensional implicit plot of the restoring force *versus* the displacement. Since niether of the upper plots show a periodic steady-state, no limit cycle is observed in the lower plot and, therefore, the particular Class I system in Equation (5) is said to be passive.

**Figure 3:**
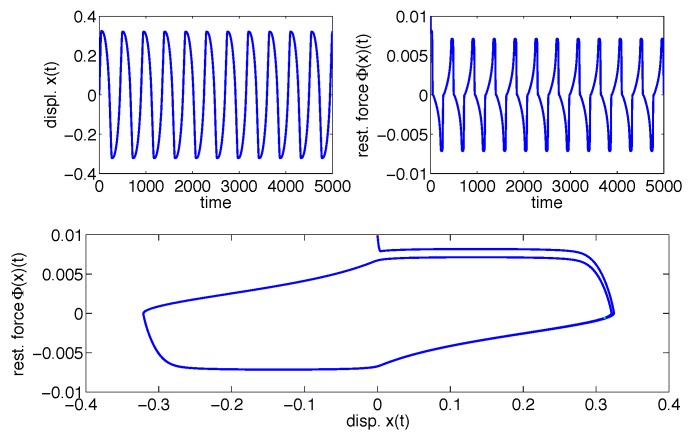
Limit cycles for a Class IV double-loop Bouc–Wen model.

Contrarily, consider now that Φ(x)(t) belongs to Class IV. Thus, for instance, A=-2, β=3, λ=-4, γ=1 and n=1. The upper left plot in Figure 3 shows the displacement x(t) of the system described in Equation (5) *versus* the time, the upper right plot shows the restoring force Φ(x)(t) *versus* the time and, finally, the lower plot shows the two-dimensional implicit plot of the restoring force *versus* the displacement. In this case, however, both upper plots show a periodic steady-state, and a limit cycle is observed in the lower plot. Therefore, the Class IV model is not physically consistent, as stated.

## 3. Passivity

In this section, the Class I double-loop Bouc–Wen model in Equations (1)–(4) is shown to be passive. The relationship between Lyapunov stability and passivity is very close [22]. This connection can be determined by using a storage function as a Lyapunov function. More precisely, passivity is the property describing that any storage energy in a system is less than or equal to the energy supplied to this system from external sources. Therefore, the connection between passivity and Lyapunov stability increases the interest in passivity. However, passivity is not only linked to Lyapunov stability, since there are also some elements that connects optimality and passivity [23]. Finally, passivity can also be considered as a particular case of dissipativity [24]. Passivity has been considered in recent years in many different areas: in electrical networks, for instance, passitivy implies that the network consists of passive elements so that the network does not produce energy. In mechanics, passivity is also associated to energy dissipation.

### 3.1. Definition

As previously stated, the passivity of a system is related to energy dissipation. A system is passive if it does not generate energy but only dissipates it. Therefore passive systems do not store more energy than that supplied to it by external sources. With this in mind, we can then define the passivity of a dynamical system in this way:

**Definition 2**  (passive system, [12]).* Consider a dynamical system with input u and output y given by:*

$$\begin{array}{cc} \dot{h}= & F(h,u)\\ y= & G(h,u) \end{array}$$

*where*

$$\begin{array}{c} F:R^{n}\times R\to R^{n} \end{array}$$

*is locally Lipschitz,*

$$\begin{array}{c} G:R^{n}\times R\to R \end{array}$$

*is continuous and*

$$\begin{array}{cc} F(0,0) & =0\\ G(0,0) & =0 \end{array}$$

The system is said to be passive if there exists a continuously differentiable positive semi-definite function V(x), called the *storage function*, such that

$$\dot{V}=\frac{\partial V}{\partial h}F\left(h,u\right)\leq yu,\;\forall \left(h,u\right)\in R^{n}\times R$$

### 3.2. Passivity of the Double-Loop Bouc–Wen Model Using Acceleration Information

In this section we present the passivity proof of the Class I double-loop Bouc–Wen model in Equations (1) and (2). Recall that Class I double-loop Bouc–Wen models are those that satisfy A±γ>0, β+λ>0, β-λ≥0.

**Theorem 2.**  *Consider the Class* I *double-loop Bouc–Wen model using acceleration information given by:*

$$\begin{array}{cc} \dot{z} & =D^{-1}(A\dot{x}-\beta |\dot{x}{\left||z\right|}^{n-1}z-\lambda \dot{x}{\left|z\right|}^{n}+\gamma \mathrm{sgn}\left(\ddot{x}\right)|\dot{x}\left|\right) \end{array}$$

*whose model parameters satisfy* A±γ>0, β+λ>0, β-λ≥0. *Then, the model is passive with respect to the storage function* $W_{a}$ *defined in Equation* (14).

**Proof of Theorem 2.**  Consider the Class I double-loop Bouc–Wen model given by:

$$\begin{array}{cc} \dot{z} & =D^{-1}(A\dot{x}-\beta |\dot{x}{\left||z\right|}^{n-1}z-\lambda \dot{x}{\left|z\right|}^{n}+\gamma \mathrm{sgn}\left(\ddot{x}\right)|\dot{x}\left|\right) \end{array}$$

and the map given by Equations (1) and (2).

Let the model of Equations (1) and (2) be rewritten as:

(6) $$\begin{array}{cc} \dot{x}= & u \end{array}$$

(7) $$\begin{array}{cc} \dot{z}= & D^{-1}\left(Au-{\beta |u||z|}^{n-1}z-{\lambda u|z|}^{n}+\gamma \mathrm{sgn}\left(\dot{u}\right)\left|u\right|\right) \end{array}$$

(8) $$\begin{array}{cc} y= & \alpha kx+\left(1-\alpha \right)Dkz \end{array}$$

This model can be seen as a nonlinear system whose input is the velocity $\dot{x}=u\left(t\right)$ and whose output is $y\left(t\right)=\Phi_{\text{acc}}\left(x\right)\left(t\right)$. The displacement and the variable z(t) can be seen as state variables. Consider Equation (7) and recall the constraints imposed on *A*, *β*, *γ* and *λ*:

$$\begin{array}{cc} A\pm \gamma & >0\\ \beta +\lambda & >0\\ \beta -\lambda & \geq 0 \end{array}$$

Thus, by multiplying both sides of Equation (7) by Dz we obtain:

Dzz˙=Ax˙-β|x˙||z|n-1z-λx˙|z|n+γsgn(x¨)|x˙|z=Azx˙-β|x˙||z|n+1-λx˙|z|nz+γsgn(x¨)|x˙|z(9)≤Azx˙+γsgn(x¨)|x˙|z+(|λ|-β)|x˙||z|n+1

Recall that β>0 and note that β+λ>0 and β-λ≥0 imply that |λ|-β<0. Therefore, Equation (9) can be written as:

Dzz˙≤A+γsgn(x¨)sgn(x˙)zx˙(10)=(A±γ)zx˙

Thus, from Equation (10):

(11) $$z\dot{x}\geq \frac{Dz\dot{z}}{A\pm \gamma }$$

Using Equations (8) and (11) yields:

$$\begin{array}{cc} yu= & \alpha kxu+(1-\alpha )Dkzu\\ = & \alpha kx\dot{x}+\left(1-\alpha \right)Dkz\dot{x}\\ \geq & \alpha kx\dot{x}+\left(1-\alpha \right)Dk\frac{Dz\dot{z}}{A\pm \gamma }\\ = & \alpha kx\dot{x}+\frac{\left(1-\alpha \right)D^{2}k}{A\pm \gamma }z\dot{z}\\ = & 2C_{a_{1}}x\dot{x}+2C_{a_{2}}z\dot{z}={\dot{W}}_{a} \end{array}$$

where

(12) $$\begin{array}{cc} C_{a_{1}}= & \frac{\alpha k}{2}>0 \end{array}$$

(13) $$\begin{array}{cc} C_{a_{2}}= & \frac{\left(1-\alpha \right)D^{2}k}{2(A\pm \gamma )}>0 \end{array}$$

(14) $$\begin{array}{cc} W_{a}= & C_{a_{1}}x^{2}+C_{a_{2}}z^{2} \end{array}$$

Thus, the double-hysteretic system with acceleration information is passive with respect to the storage function $W_{a}$, as we wanted to prove. □

### 3.3. Passivity Proof of the Double-Loop Bouc–Wen Model Using Position Information

As in Section 3.2, in this section we present the passivity proof of the Class I double-loop Bouc–Wen model in Equations (3) and (4). Recall again that Class I double-loop Bouc–Wen models are those that satisfy A±γ>0, β+λ>0, β-λ≥0.

**Theorem 3.**  *Consider the Class* I *double-loop Bouc–Wen model using position information given by:*

$$\begin{array}{cc} \dot{z} & =D^{-1}(A\dot{x}-\beta |\dot{x}{\left||z\right|}^{n-1}z-\lambda \dot{x}{\left|z\right|}^{n}+\gamma \mathrm{sgn}\left(x\right)|\dot{x}\left|\right) \end{array}$$

*whose model parameters satisfy* A±γ>0, β+λ>0, β-λ≥0. *Then, the model is passive with respect to the storage function* $W_{p}$ *defined in Equation* (21).

**Proof of Theorem 3.**  Consider now a physical system with a hysteretic component that can be represented by the map given by Equations (3) and (4).

The pasivity proof is similar to that of the system with acceleration information and is summarized as follows.

Let the model of Equations (3) and (4) be rewritten as:

(15) $$\begin{array}{cc} \dot{x}= & u \end{array}$$

(16) $$\begin{array}{cc} \dot{z}= & D^{-1}\left(Au-{\beta |u||z|}^{n-1}z-{\lambda u|z|}^{n}+\gamma \mathrm{sgn}\left(x\right)\left|u\right|\right) \end{array}$$

(17) $$\begin{array}{cc} y= & \alpha kx+\left(1-\alpha \right)DKz \end{array}$$

Thus, by multiplying both sides of Equation (16) by Dz we obtain:

Dzz˙=Ax˙z+γsgn(x)|x˙|z-β|x˙||z|n+1-λx˙|z|nz(18)≤(A±γ)zx˙

From Equations (15), (17) and (18) we have that:

$$\begin{array}{cc} yu= & \alpha kx\dot{x}+\left(1-\alpha \right)Dkz\dot{x}\\ \geq & \alpha kx\dot{x}+\left(1-\alpha \right)\frac{\left(1-\alpha \right)D^{2}k}{A\pm \gamma }z\dot{z}\\ = & 2C_{p_{1}}x\dot{x}+2C_{p_{2}}z\dot{z}={\dot{W}}_{p} \end{array}$$

where

(19) $$\begin{array}{cc} C_{p_{1}}= & \frac{\alpha k}{2}>0 \end{array}$$

(20) $$\begin{array}{cc} C_{p_{2}}= & \frac{\left(1-\alpha \right)D^{2}k}{2(A\pm \gamma )}>0 \end{array}$$

(21) $$\begin{array}{cc} W_{p}= & C_{p_{1}}x^{2}+C_{p_{2}}z^{2} \end{array}$$

Thus, the double-hysteretic system with position information is passive with respect to the storage function $W_{p}$. □

## 4. Conclusions

The Bouc–Wen hysteresis model is widely employed to mathematically represent the dynamical behavior of several physical devices, materials and systems such as magnetorheological dampers, lanthanide zirconium or aluminum oxides, electromagnetic servo motors, mechanical structures or biomedical systems. However, these mathematical models must account for different properties such as the bounded-input bounded-output stability, asymptotic motion, thermodynamic admissibility or passivity in order to be physically consistent with the systems they represent.

The passivity of a system is related to energy dissipation. That is, a system is passive if it does not generate energy but only dissipates it. In this paper we have studied the passivity of a particular class of two double-loop Bouc–Wen models that uses position and acceleration information. It has been shown that both double-loop Bouc–Wen models are passive for a particular set of model parameters set and thus they are physically consistent and suitable for real systems.
